# Social Experiments in the Mesoscale: Humans Playing a Spatial Prisoner's Dilemma

**DOI:** 10.1371/journal.pone.0013749

**Published:** 2010-11-12

**Authors:** Jelena Grujić, Constanza Fosco, Lourdes Araujo, José A. Cuesta, Angel Sánchez

**Affiliations:** 1 Grupo Interdisciplinar de Sistemas Complejos (GISC), Departamento de Matemáticas, Universidad Carlos III de Madrid, Leganés, Madrid, Spain; 2 Grupo de Procesamiento de Lenguaje Natural (NLP and IR), Departamento de Lenguajes y Sistemas, UNED, Madrid, Spain; 3 Instituto de Ciencias Matemáticas, CSIC-UAM-UC3M-UCM, Cantoblanco, Madrid, Spain; 4 Instituto de Biocomputación y Física de Sistemas Complejos (BIFI), Universidad de Zaragoza, Zaragoza, Spain; University of Zurich, Switzerland

## Abstract

**Background:**

The evolutionary origin of cooperation among unrelated individuals remains a key unsolved issue across several disciplines. Prominent among the several mechanisms proposed to explain how cooperation can emerge is the existence of a population structure that determines the interactions among individuals. Many models have explored analytically and by simulation the effects of such a structure, particularly in the framework of the Prisoner's Dilemma, but the results of these models largely depend on details such as the type of spatial structure or the evolutionary dynamics. Therefore, experimental work suitably designed to address this question is needed to probe these issues.

**Methods and Findings:**

We have designed an experiment to test the emergence of cooperation when humans play Prisoner's Dilemma on a network whose size is comparable to that of simulations. We find that the cooperation level declines to an asymptotic state with low but nonzero cooperation. Regarding players' behavior, we observe that the population is heterogeneous, consisting of a high percentage of defectors, a smaller one of cooperators, and a large group that shares features of the conditional cooperators of public goods games. We propose an agent-based model based on the coexistence of these different strategies that is in good agreement with all the experimental observations.

**Conclusions:**

In our large experimental setup, cooperation was not promoted by the existence of a lattice beyond a residual level (around 20%) typical of public goods experiments. Our findings also indicate that both heterogeneity and a “moody” conditional cooperation strategy, in which the probability of cooperating also depends on the player's previous action, are required to understand the outcome of the experiment. These results could impact the way game theory on graphs is used to model human interactions in structured groups.

## Introduction

The mechanisms underlying the emergence of cooperation are as yet an unsolved puzzle. Understanding them is key because all major transitions in evolution involve the spreading of some sort of cooperative behavior [1], bringing about a higher level of complexity. But cooperation is costly and amenable to free-riding by defectors, so a mechanism that favors the assortative interaction of cooperators is required to transform cooperation into the most profitable strategy [2]. As cooperation is observed in biological and social systems alike, different mechanisms stressing particular aspects of these different disciplines have been proposed to explain cooperation [3]. One such route is the existence of a (social, spatial, geographical) structure that determines the interactions among individuals in the population [4], [5]. A great deal of research has been devoted to understand the effects of population structure on cooperation [6], [7]. Most of those works have studied the Prisoner's Dilemma (PD) as the paradigmatic framework in which cooperation is the socially desirable outcome but is dominated by the rationality of defection [4], [8], [9]. However, this large body of research has not been conclusive because model details, chiefly the type of spatial structure and the evolutionary dynamics [7], [10], lead to dramatic differences between predictions. Therefore, experimental work beyond the large body of results on the PD on unstructured populations [11], [12] is needed to ascertain both the relevance of the population structure and the types of dynamics that are actually at work in real situations.

To progress towards answering these two questions we have focused on the pioneering model studied by Nowak and May [5]. They simulated a set of agents located on a square lattice, playing a PD with their Moore neighborhood (i.e., playing a PD with each of their eight neighbors, but using the same strategy in all of them), and showed that when they imitated the behavior of their neighbor who obtained the largest payoff in the previous round (including themselves), cooperation could thrive even under very adverse choices of the payoffs. Inspired by this, we have carried out an experiment with human subjects playing a PD on a sizeable network, with a setup as close as possible to the one of Nowak and May's simulations. In this respect, it is important to note that in those simulations agents do not have memory and update their strategies with a specific, fixed rule, whereas we are implementing the same system with humans. It is clear that the rules used by humans are unknown a priori (they are not instructed to follow any particular rule), hence the goal of the experiment is precisely to unveil the way they behave.

Specifically, 169 volunteers were located on a (virtual) 13
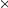
13 square lattice with periodic boundary conditions, on which they were able to interact anonymously. This is by far the largest experiment of this kind ever carried out, and organizational difficulties as well as increasing costs prevent from working with much larger systems. In fact, to our knowledge, experimental work on this issue has been conducted only recently and on networks an order of magnitude smaller [13], [14], and only one has addressed the questions we are interested in here, using 4
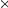
4 lattices [15]. The question of the size of the network is very important, because the putative mechanism leading to the emergence of cooperation [10], [16] is the appearance of clusters of cooperators. Cooperator clustering can be difficult to observe in small systems, hence the necessity of studying larger ones and, in addition, for times as long as possible [17].

## Results

### Experimental setup

In our experiment, volunteers played a 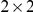 PD game with each of their eight neighbors (Moore neighborhood) taking only one action, either to cooperate (C) or to defect (D), the action being the same against all the opponents. The resulting payoff was calculated by adding all eight interaction payoffs. Payoffs of the PD game were set to be 7 cents of a euro for mutual cooperation, 10 cents for a defector facing a cooperator, and 0 cents for any player facing a defector (weak PD [5]). With this choice (a cooperator and a defector receive the same payoff against a defector) defection is not a risk dominant strategy, which enhances the possibility that cooperation emerges. In addition, to avoid framing effects, the two actions were always referred to in terms of colors (blue for C and yellow for D), and the game was never referred to as PD in the material handed to the volunteers. This notwithstanding, players were properly informed of the consequences of choosing each action, and some examples were given to them in the introduction. After every round players were given the information of the actions taken by their neighbors and their corresponding payoffs.

The full experiment consisted of three parts: experiment 1, control, and experiment 2. In experiment 1 players remained at the same positions in the lattice with the same neighbors throughout the experiment. In the control part we removed the effect of the lattice by shuffling players every round. Finally, in experiment 2 players were again fixed on a lattice, albeit different from that of experiment 1. On the screen players saw the actions and payoffs of their neighbors from the previous round, who in the control part were different from their current neighbors with high probability. All three parts of the experiment were carried out in sequence with the same players. Players were also fully informed of the different setups they were going to run through. The number of rounds in each part was randomly chosen between 40 and 60 in order to avoid players knowing in advance when it was going to finish, resulting in 47, 60, and 58 rounds for experiment 1, control, and experiment 2, respectively.

### Global cooperative behavior

**Figure 1:**
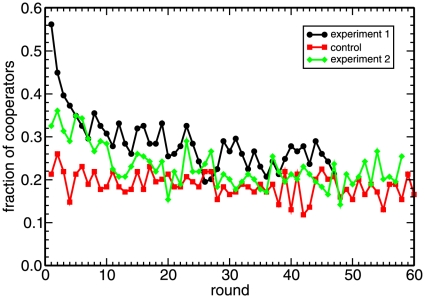
The cooperation level declines to a low but non-zero level. Fraction of cooperators in every round of the three parts of the experiment (in the first and the last ones players remain in the same node of the lattice along the whole experiment, whereas in the control part players are shuffled every round). Players are arranged in a 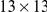 lattice with periodic boundary conditions, and play a PD game with each of its 8 surrounding neighbors in the lattice. With the notation C for cooperation, D for defection, and 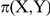 for the payoff obtained by a player who plays X against an opponent who plays Y, the payoff matrix of each of these PD games is 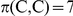 cents of a euro, 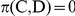 cents, 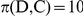 cents, and 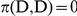 cents. These payoffs conform a *weak* PD game —the most favorable to promote cooperation— because 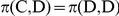. This setup is entirely similar to that of Nowak and May's simulations [5] except for the size of the lattice (simulations are performed on 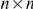 lattices, with 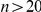) and the lack of self-interactions (see [17] for further comments).

We begin the presentation of the results of our experiment by discussing the first issue, namely the global cooperation level. Figure 1 represents the total percentage of cooperative actions in every round of the three parts of the experiment. Experiment 1 begins with a very large percentage of cooperation, above 50%, that rapidly decays to reach a more or less constant level after some 25 rounds. Experiment 2 exhibits the same behavior, but the initial cooperation level is much lower, a 32%, and the transient shorter. On the contrary, the control part shows a constant fraction of cooperative actions, fluctuating around 20%. This is a clear indication that players did realize that the fact that neighbors changed after every round made it hopeless to try to achieve a mutually profitable environment, which they did attempt to establish at the beginning of experiment 1 (particularly so) and experiment 2. On the other hand, after the initial transient, the amounts of cooperation observed in the two experiments and in the control part coincide approximately, showing that the existence of a fixed lattice structure has little influence on the players' asymptotic behavior.

Our conclusion that the lattice has little influence for the global cooperation level and our observed results are in good agreement with those reported by Traulsen *et al.* [15], although in their case they also observe high initial cooperation levels in the well-mixed case, most likely because in their setup these players were beginning their participation without prior experience. We note also that their experiment is shorter in time than ours, with a duration comparable to the length of our transient (they do not observe a stationary state, as we do, as noted also in [17]). In spite of that, it appears that their asymptotic value for cooperation is compatible with the 20% value we found. On the other hand, the differences between the results of experiments 1 and 2 cannot be attributed to the different distributions of players on the lattice: A learning process has occurred that has led players to use a better defined strategy in experiment 2. This is not only evident in the shorter transient period and the lower starting level of cooperation in experiment 2 compared to experiment 1, but it also shows up in many other features that we will be commenting on in the remaining of this article.

### Testing the “imitate-the-best” strategy

Our experiment has been set up to mimic Nowak and May's simulations as close as possible. As the system sizes considered in [5] were larger than our experimental lattice, we have repeated their simulations on a 13
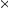
13 lattice with the payoffs of the experiment. We also used the same update rule, “imitate-the-best” —copying the action of the neighbor that performed the best provided it was better than their own—. The results show that the asymptotic level of cooperation is either 0 or a large value close to 1, depending on the initial condition, while an outcome with the level of cooperation observed in the experiment is never found. This suggests that either players do not update their actions with an imitate-the-best rule, or memory effects, absent in [5], are important —or both. We will analyze the behavior of the players in terms of their previous actions and those of their neighbors in the next section. Presently, we will check to what extent imitation plays a role in our experiment. To that purpose we have computed the fraction of actions that can be interpreted as imitation of the best action in the neighborhood along the experiment, yielding the values 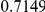 for experiment 1 and 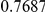 for experiment 2. In spite of their being both above 70% one should bear in mind that there are only two actions to choose from and pure chance may be mistaken for imitation. To ascertain the statistical significance of these values we applied a non-parametric bootstrap [18] method, consisting of performing a thousand random shufflings of the positions of the players while keeping their sequences of actions during the experiments, and computing the corresponding fractions of imitation. This provides the empirical probability distributions of the null hypothesis “imitation is due to chance”. The mean values of these distributions are 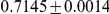 for experiment 1 and 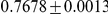 for experiment 2, and values larger than the one we find can be obtained with probability 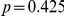 in experiment 1 and 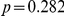 in experiment 2. This proves that the observed imitation is not significantly different from the apparent imitation yielded by pure chance. This result, which is consistent with the low level of cooperation observed (players using imitate-the-best should lead the system to higher cooperation) and with the responses to the questionnaires at the end of the experiment (no one claimed to have imitated the best neighbor), makes it plausible to conclude that imitate-the-best is not an appropriate explanation of players' behavior (although strictly speaking, this statistical analysis does not allow us to definitely rule out this strategy).

### Analysis of players' strategies during the experiment

**Figure 2:**
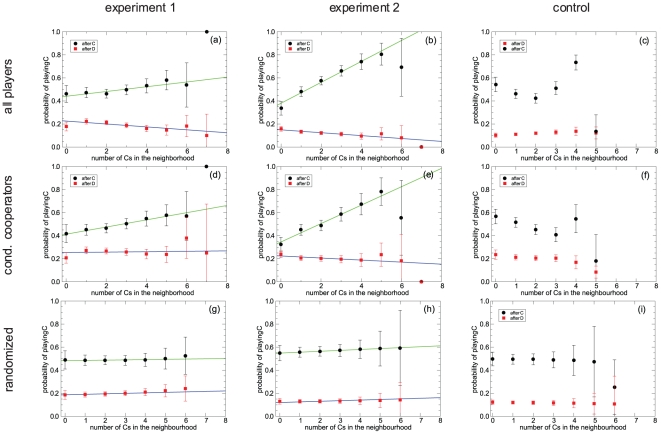
Context-dependent behavior depends also on the player's previous action or “mood”. Probabilities of cooperating after playing C or D, conditioned to the context (number of cooperators in the previous round). Panels (a)–(c) show results for all players, whereas panels (d)–(f) show results for the group of players referred to as conditional cooperators. Panels (a) and (d) correspond to results of experiment 1, panels (b) and (e) to experiment 2, and panels (c) and (f) to the control experiment. The parameters of the linear fits can be found in Table 1. The plots demonstrate that there is a strong dependence on the context for players that cooperated in the previous round (i.e., were in a “cooperative mood”), the cooperation probability increasing rapidly as a function of the number of cooperative neighbors in a manner similar to the conditional cooperators found by Fischbacher *et al.* [19]. However, after having defected, players behave in a manner that shares features of exploiting behavior, cooperating with equal or less probability as the number of cooperators in their neighborhood increases. The very different behavior of players in the control experiment illustrates that conditional cooperation arises as a direct reciprocity effect —which is pointless if neighbors change every round. The conditioning of cooperation to the previous round is different in both experiments, which provides a strong indication that players learnt to play as the experiment proceeded, moody conditional cooperation being more clearly observed in the plots corresponding to experiment 2. Finally, panels (g)–(i) show the probabilities of cooperating after playing C or D, conditioned to the context (number of cooperators in the previous round), averaged over 1000 random shufflings of players in the lattice. Panel (g) corresponds to experiment 1, panel (h) to experiment 2, and panel (i) to the control experiment. The results show that there is no dependence on the context, proving that the dependence revealed in panels (a)–(f) is statistically significant.

**Figure 3:**
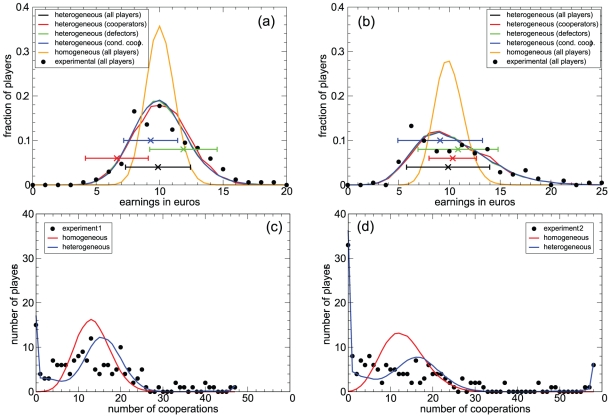
The heterogeneous model reproduces the earning and cooperation histograms and supports the coexistence of different types of players. Panels (a) and (b): Histograms of earnings in simulations of the heterogeneous model, for all players aggregated (black line, hidden by the blue line) as well as for the three basic types of players: pure and mostly cooperators (red line), pure and mostly defectors (green line), and conditional cooperators (blue line); histograms of earnings in simulations of the homogeneous model (orange line); and experimental histograms of earnings for all players aggregated (black dots). Results are presented for both experiment 1 (a) and experiment 2 (b). Simulations results are averages over 1000 runs. Crosses (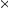) represent the mean earnings in the real experiments (their Y coordinate is arbitrary). Error bars span two standard deviations. Clearly, simulations of the homogeneous model do not fit the experimental data, thus supporting the introduction of the heterogeneous model. There is a reasonable consistency between experimental results and numerical simulations for the heterogeneous model, more so in experiment 2, where players are supposed to be playing with a better defined strategy. In experiment 1, the longer cooperative transient makes defection a more favorable strategy. The fact that the histograms for the different kinds of players are indistinguishable supports the coexistence of strategies, as there is no real incentive (on average) to switch from one strategy to any other. Panels (c) and (d): Number of players who cooperate a given number of rounds, both for experiment 1 (c) and experiment 2 (d). The experimental results are plotted together with the results of simulations with the homogeneous and the heterogeneous models, averaged over 1000 realizations. Once again, the homogeneous model is not able to reproduce the experimental results.

To make further progress towards clarifying the question of the dynamics of strategies, we considered as an alternative strategy update rule the possibility that players react to the number of cooperative neighbors (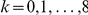) they observed in the previous round (henceforth a *context*), i.e., we assume that they have one-step memory. This is a reasonable assumption in view that questionnaires suggest that players take into account what their neighbors do. Furthermore, Traulsen *et al.* [15] briefly report that cooperative actions are more frequent in more cooperative environments. Therefore, we specifically computed from the experimental data the average frequency with which players cooperated, conditioned to both their previous action and their context, and made linear fits to these frequencies [Figures 2(a) and (b) and Table 1]. The first observation is that players' reactions to the context depend strongly on the past action of the focus player, something that to our knowledge has never been reported. The significance of this result can be assessed by comparing with the result obtained averaging over a thousand shufflings of the players in the lattice [Figures 2(g) and (h)], which show no dependence on the context. On the other hand, the differences observed in the fits of the two experiments provide another hint that players are using a better defined strategy in experiment 2, after having “learnt” in the two previous phases of the experiment. Using these fits as a model (henceforth *homogeneous model*), we made simulations in a 13
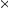
13 lattice in which all players react according to these rules, with an initial condition similar to the one found in the experiments. This model is able to reproduce the observed asymptotic level of cooperation in both experiments, predicting an asymptotic value of 28% for experiment 1 and of 22% for experiment 2, but fails to reproduce other features. For instance, it leads to a histogram of total earnings much narrower than the experimental one, and the distribution of fractions of cooperative actions among players reveals that it does not capture a significant fraction of stubborn defectors and cooperators that appear in the experiment (see Figure 3).

**Table 1:** Linear fits to the probabilities of cooperating as a function of the context.

type of data	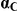	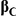	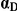	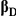
exp. 1, all players	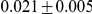	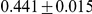	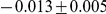	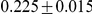
exp. 2, all players	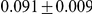	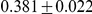	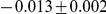	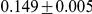
exp. 1, cond. coop.	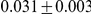	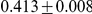	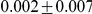	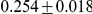
exp. 2, cond. coop.	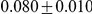	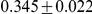	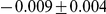	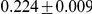

Fits are defined by 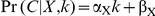, where 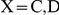 is the player's action in the previous round and 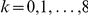 is the number of cooperators in the neighborhood in the previous round.

We then tried to distinguish different kinds of behavior shown by players. First we found a sizeable number of *pure defectors*, as well as a few *pure cooperators*, in all three stages of the experiments —i.e., players who always defected/cooperated irrespective of the actions of their neighbors. Taking these individuals out, we still were able to classify the remaining players into three groups: *Mostly defectors* (people who defected more than 2/3 of the times in any context), *mostly cooperators* (cooperated more than 2/3 of the times in any context), and generalized conditional cooperators (players who seem to react to the context as before), which we hereafter refer to as *moody conditional cooperators* —indicating that their propensity to cooperate depends on their previous action, or “mood”. Their amounts are listed in Table 2 and we have checked that this classification is consistent with the answers that players provided in their questionnaires. It is remarkable that the classification is very similar to the one reported by Fischbacher *et al.* [19] in public goods experiments, and confirmed in subsequent papers (see, e.g., [20] for a review and [11] for a general review about public goods experiments), even if they do not report the “moody” behavior of conditional cooperators. This is an important feature of their behavior because, as can be seen in Figures 2(a) and (b), the probability that a moody conditional cooperator cooperates after having defected in the previous round turns out to be slightly non-increasing as a function of the number of cooperators in the context.

**Table 2:** Evidence for heterogeneity in the behavior of the population.

type of player	experiment 1	control	experiment 2
pure cooperators	1	1	6
mostly cooperators	2	2	3
conditional cooperators	125	92	91
mostly defectors	26	36	36
pure defectors	15	38	33

Frequency of the different types of players in the three parts of the experiment. Mostly defectors are people who defected more than 2/3 of the times in any context, mostly cooperators are those who cooperated more than 2/3 of the times in any context, and conditional cooperators follow the strategy described in the main text.

It is worthwhile to compare the behavior of conditional cooperators in the two experiments [either Figures 2(a) and (b) or 2(d) and (e)] and in the control part [Figure 2(c) or 2(f), respectively]. The different behavior that can be observed strongly suggests that this strategy arises as a result of direct reciprocity. Whereas in the two experiments conditional cooperators who cooperated in the previous action cooperate more the more neighbors cooperate, it is quite the opposite in the control experiment. Indeed, it makes no sense to reciprocate or retaliate in this control experiment because the recipients of your action are —with high probability— no more your previous opponents.

### Cooperator clustering

Once we have a classification of the players, we are in a position to address another issue about the lack of global cooperative behavior, namely the assortment or clustering of cooperators. The low level of cooperation we observe is in agreement with the fact that cooperative players —i.e., players whose actions are always or almost always cooperative— do not cluster in space even if they are initially a majority, as in experiment 1. Interestingly though, the few cooperators in experiment 2 are somewhat clustered, and in both experiment 1 and 2, defectors show a slight anti-clustering trend: This can indeed be seen in Table 3, where we collect the average number of neighbors of the same type for the three types of players (pure and mostly cooperators, pure and mostly defectors, and conditional cooperators), as obtained from the experimental data. This average is computed, for each type of player, as the sum of pairs of neighbors of the given type divided by the number of players of that type. We resorted again to non-parametric bootstrapping to assign significance to those values, computing the average number of neighbors of the same type in a thousand random shufflings of players. The experimental values are always within the confidence interval of the null model, except for a few cases (in boldface in Table 3) that are particularly important because they suggest some cooperator clustering as well as some defector anti-clustering, precisely the cooperation fostering mechanism put forward by theoretical models. It would nevertheless be bold to speak about clustering of cooperators when the largest number of them we observe (that of experiment 2) is just nine.

**Table 3:** Average number of neighbors of the same type.

type	experiment 1			experiment 2		
	exper.	mean	SD	exper.	mean	SD
cooperator	0.0000	0.0946	0.2383	**1.3333**	0.3905	0.2740
cond. coop.	5.8560	5.9048	0.0819	4.1758	4.2855	0.1438
defector	**1.6585**	1.9163	0.2353	**2.9565**	3.2404	0.1823

The column *exper.* lists the average number of neighbors of the same type for the three types of players, computed, for each type of player, as the sum of pairs of neighbors of the given type divided by the number of players of that type. The columns *mean* and *SD* list the means and standard deviations of the values obtained in 1000 random shufflings of players.

### Heterogeneous model

In order to assess the validity of our understanding of the players' behavior we designed a new model implementing heterogeneity by starting from the same amounts of each of the five types of players (the model is referred to as *heterogeneous model*). In the simulations every player behaves according to her type, and for the generalized conditional cooperators we employed a model similar to the homogeneous one, but this time computing the average probabilities only for conditional cooperators [see Figures 2(d) and (e) and Table 1]. This heterogeneous model succeeds in reproducing even the features that the homogeneous model does not capture. To begin with, the global cooperation level is 28% for experiment 1 and 23% for experiment 2, in agreement with the experimental results. Furthermore, Figure 3(a) and (b) shows a comparison of the histogram of earnings, for all players aggregated and separated by types, as obtained from the two models (homogeneous and heterogeneous) and from the experiment. We can observe that experimental data are consistent with the simulations of the heterogeneous model, whereas the homogeneous model deviates from the experimental results (typically, as we already mentioned, it has a noticeably narrower distribution of earnings). This picture also shows that the distribution of earnings is the same for all kinds of players, clearly in the simulations but also in the experimental data, mainly in experiment 2. The slight advantage of defectors in experiment 1 is surely due to the longer cooperative transient. This advantage disappears in experiment 2, where players are supposed to have learnt and to be using a more definite strategy. We note that the fact that payoffs are very similar for the different strategies supports their coexistence, as there is no real incentive (on average) to switch between them. Interestingly, a similar result was found in experiments on modified public goods games by Kurzban and Houser [21]. A further evidence in favor of the heterogeneous model is revealed by the histogram of cooperative actions occurred in both experiments [Figure 3(c) and (d)]. The homogeneous model shows a Gaussian-like peak, whereas the heterogeneous model shows a more widespread distribution, closer to the experimental one.

### Alternative interpretations of players' strategies

The fact that Figure 2 reveals that the probability of cooperating after having defected in the previous round is both low and independent on the context, might suggest that the strategy actually employed by conditional cooperators is a version of GRIM. GRIM is a strategy of the so called “trigger” type, first introduced by Friedman [22]. This strategy amounts to cooperating until disappointment (by the lack of cooperation of the partners), and defecting from then on. Thus defined, GRIM plays an important role for proving theoretical results in game theory (see, e.g., [23], [24]). For our present purposes, let us note that if all or a majority of agents use this strategy, it is clear that as soon as one defects, a cascade of permanent retaliation is initiated until full defection dominates the system. This is the reason why in the famous experiments by Axelrod about the PD game GRIM did not perform very well (cf. [4], where GRIM is referred to as FRIEDMAN). In our experiment we observe a background of cooperative actions near 20%, but perhaps players are using a weaker version of GRIM in which the final defection is ‘noisy’ in the same percentage. Alternatively, players could be progressively switching from an initial conditional cooperative strategy to a more defective strategy through some learning process (see, e.g., [12] for a review of the different learning processes that could be at work). In both cases the result found in Figure 2 for the probability of cooperating after having cooperated in the previous round would just be a consequence of the actions taken by these players during the transient, in the first rounds of both experiments, and the asymptotically surviving strategy would be noisy defection, regardless of the context.

**Figure 4:**
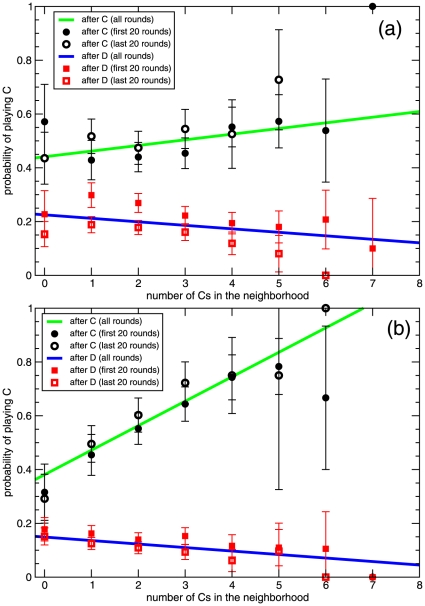
Conditional cooperators' strategies (almost) do not change over time. Same as Figure 2 for the case with all players in experiments 1 (a) and 2 (b). Straight lines are the fit appearing in Figure 2, whereas points are the strategies as obtained only from the first 20 rounds (full symbols) and only from the last 20 rounds (empty symbols). The strategies are statistically the same for experiment 2, and for the experiment 1 after having played C in the previous round. After having played D in the previous round in experiment 1, the probability of cooperating noticeably decreases over time down to a value compatible with that observed in experiment 2.

To test this alternative explanation, we have carried out an analysis of the conditional strategies at different times during the game. If any of these two strategies is at use, this analysis should reveal a change in the probabilities shown in Figure 2 over time; in particular, we should observe a decay of the probability of cooperating after having cooperated in the previous round. We do not have enough statistics to test the strategies every round of the game, but we can do it along two different intervals: in the first 20 rounds and in the last 20 rounds. Did the player use any GRIM-like strategy, either as such or through a learning process, the results of the analysis in these two periods should be different, as least as far as cooperative strategies are concerned. In Figure 4 we show the results of this analysis. We do not observe any significant change in the results for experiment 2, and for experiment 1 we only appreciate a small decay of the probability of cooperating after having defected. These results rule out the interpretation of players' strategies as GRIM or as ‘learning-to-defect’, with the exception, in this last case, of the small effect just pointed out. Our result is in agreement with recent experimental findings [25] that in an infinitely repeated PD game GRIM explains some of the data, but its proportion is not statistically significant. It seems that during experiment 1 the probability that a player restores cooperation gets adjusted as time passes, decreasing towards values compatible with the stable value found in experiment 2. On the other hand, there is, of course, the difference in the cooperative strategy between both experiments, also attributable to some kind of learning. Particularly interesting is the stability of the strategies along experiment 2, consistent with the idea that players had a more precise idea of how to play in this second experiment than they had in the first one.

## Discussion

The large size of our experimental setup and the data analysis presented above allow us to contribute to the two questions we wanted to address. First, we have observed that the existence of a lattice giving structure to a population playing PD does not lead to an increase of the cooperation level, even if as in our case the PD is weak. Thus, subjects behave as if they were playing a repeated public goods game, the fact that the game in which a player is involved overlaps with those of their neighbors having very little influence on the observed asymptotic level of cooperation. Second, regarding the manner in which people update their strategies, we have not found evidence in favor of imitate-the-best behavior, in agreement with the analysis in [14], [15]. These two observations imply in turn that the model simulated in [5] does not describe our experiment with human subjects —albeit it may of course be applicable in many other instances such as, e.g., experiments with bacteria. We then analyzed the way subjects behaved by considering that they might be influenced by the previous actions of their neighbors. This analysis has allowed us to make some progress in understanding human behavior, reaching two important conclusions about individual learning models. The first one is that there is a large degree of heterogeneity, with an important fraction (25–45%) of players sticking to a strategy of (almost) always defect or cooperate. This is a crucial observation because the experimental results are not recovered unless those individuals are included in the modeling. The analysis of the total earnings of players also suggests that this heterogeneity can be evolutionarily stable, in the sense that all strategies are (on average) equally profitable, as observed also in [21] (some theoretical support for the evolutionary stability of a simplified model of conditional cooperation in the presence of social norms has been already provided [26]). The second conclusion is that the rest of the players are well described as moody conditional cooperators, i.e., players whose probability to choose one action depends on the amount of cooperation they observe in the previous round and their own previous action. Our clearest results, those of experiment 2, show that players have a high chance to continue cooperation (larger than 50%) if 3 or more neighbors cooperated, whereas if they had defected in the previous round, their chances to cooperate in the current one are small and slightly decreasing with the number of cooperating neighbors. This is consistent with an exploitation strategy which tries to incentive cooperation in low cooperative environments and also with a mutualistic strategy aiming at achieving better global results, something that many players claim to have done in their responses to the questionnaire. Indeed, the small resumption of cooperation at the beginning of experiment 2 as compared to the lack of it in the control indicates that a number of players hope they can restart cooperation for either of those two reasons. Our observation that the probability to cooperate depends on the context agrees with the results in [15], and improves them by identifying that this probability depends in turn on the focal individual's previous action. In addition, our values for the probabilities are also consistent with their observation of high levels of “mutation”, albeit our results provide a more intentional interpretation of these probabilities.

The results of this experiment have implications that go beyond the specific case study of PD on networks. Thus, the dependence on the player's own previous action we have found may be relevant to deepen our understanding of the conditional cooperation observed in public goods games [19], [20]. In addition, we have proposed a model that, in spite of its simplified description of heterogeneity, provides a more thorough picture of the way human subjects might behave in these experiments, as we show that apparent mutation can be also understood (at least partly) as conscious changes of behavior arising from cooperative or exploiting strategies. Indeed, for the first time to the best of our knowledge, a model is able to reproduce the observed features in the experiment, from the decline of cooperation through the earnings distributions to the coexistence of strategies. In this regard, it is worth noting that recent experiments by Fischbacher and Gächter [27] led to an explanation of the decline of cooperation in public goods games in which heterogeneity seemed to matter only at the end of the experiment. This is similar to what we have observed, in so far as our homogeneous model could also explain how cooperation evolved in time, but other features crucially required the introduction of heterogeneity. On the other hand, our observations are not consistent with a vast majority of the theoretical models of evolutionary games on graphs studied and simulated so far [6], [7]. Our experiment should therefore be a reference for future, more accurate modeling of these important social systems, as they strongly indicate that heterogeneity, that only recently has been considered in theoretical models [28]–[30], is a key ingredient to understand human behavior. This is crucial for the design of mechanisms that promote or at least support cooperation, one of the goals of this line of research. In this respect, our work points to avoiding early disappointment of the agents that leads them to a “defective mood” as an important aspect to act upon. Finally, the issue of finding an evolutionary explanation of this coexistence of strategies is a challenge which should also be addressed to understand human cooperative behavior.

## Materials and Methods

### Ethics statement

All participants in the experiment signed an informed consent to participate. Besides, their anonymity was always preserved (in agreement with the Spanish Law for Personal Data Protection) by letting them randomly choose a closed envelope containing a username which would identify them in the system and a password. Final payments were made to carriers of a given username. No association was ever made between their real names and the results. As it is standard in socio-economic experiments, no ethic concerns are involved other than preserving the anonymity of participants. This was checked and approved by the Viceprovost of Research of Universidad Carlos III de Madrid, the institution hosting the experiment.

### Description of the experiment

The experiment was carried out with volunteers chosen among students of the engineering campus of Universidad Carlos III in Leganés (Madrid, Spain). Following a call for participation, we received about 500 applications, among which we selected 225, with preference for the youngest ones and keeping a fifty-fifty ratio of male to female. On the day of the experiment, 178 volunteers showed up, and we kept 169, so that we could arrange them in a square lattice with periodic boundary conditions, by discarding the 9 latest arrivals —who were paid their 10 euros show-up fee and dismissed. All 169 selected participants were then directed to 11 computer rooms in two adjacent buildings, previously prepared by setting up cardboard panels between posts so that no participant could look at her physical neighbors (who anyway needed not be their actual neighbors in the game). They received directions in paper and also went through a tutorial on the screen, including questions to check their understanding of the game. When everybody had gone through the tutorial, the experiment began, lasting for approximately an hour and a half. The tutorial in Spanish, or an English translation of it, are available upon request.

At the end of the experiments volunteers were presented a small questionnaire to fill in. The list of questions (translated into English) was the following:

1. Describe briefly how you made your decisions in part I [Experiment 1].
2. Describe briefly how you made your decisions in part II [Control].
3. Describe briefly how you made your decisions in part III [Experiment 2].
4. Did you take into account your neighbors' actions?
5. Is something in the experiment familiar to you? (yes/no).
6. If so, please point out what it reminds you of.
7. If you want to make any comment, please do so below.

The first three questions have a clear motivation, namely to see whether (possibly some) players did have a strategy to decide on their actions. Question 4 was intended to check whether players decided on their own or did look at their environment, because only in this last case imitative or conditionally cooperative strategies make any sense. Questions 5 and 6 focused on the possibility that some of the players recognized the game as a Prisoner's Dilemma because they had a prior knowledge of the basics of game theory. The final question just allowed them to enter any additional comment they would like to make. We did not carry out a more detailed questionnaire to avoid the risk of many players' leaving it blank (the whole experiment was already very long). Immediately after finishing the questionnaire, all participants received their earnings and their 10 euros show-up fee. The total earnings of a player in the experiment ranged from 18 to 45 euros.

Software for the experiment was written in PHP 5, Javascript, and Python. There were 169 client computers running Opera in kiosk mode (to preclude players from doing anything else than playing according to the instructions) on Debian Linux. Clients communicated with a server on which Python programs were running controlling the experiment, making calculations, and storing results. Another client was monitoring the whole experiment, displaying every player and their current status.

The experiment assumes synchronous play, thus we had to make sure that every round ended in a certain amount of time. This playing time was set to 30 seconds, which was checked during the testing phase of the programs to be enough to make a decision, while at the same time not too long to make the experiment boring to fast players. If a player did not choose an action within these 30 seconds, the computer made the decision instead. This automatic decision was randomly chosen to be the player's previous action 80% of the times and the opposite action 20% of the times. We chose this protocol after testing several ones in simulations. We run simulations in lattices of several sizes, including the one we used, with two different update rules: imitate-the-best and proportional updating. At the same time, we included a fraction of players (up to 15%) who played with a different update rule. We tested the one we finally chose, along with similar ones with different probabilities of copying the previous action. We also tested several other rules. Our finding was that a fraction below 10% of these “singular” players can hardly affect the results whichever their update rule. So we decided to choose the 80–20 rule as the one which could pass more unnoticed to other players when confronted to it. During the experiment we monitored the fraction of players who actually played in each round. We found that in more than 90% of rounds no more than 4 of the choices were made by the computer. The largest number of automatic actions occurred at the end of the control part, but even then their number never goes beyond 8.
